# Different modalities of patellar management in primary total knee arthroplasty: a Bayesian network meta-analysis of randomized controlled trials

**DOI:** 10.1186/s13018-024-04546-w

**Published:** 2024-01-17

**Authors:** Lun Liu, Juebei Li, Yunlu Wang, Xiyong Li, Pengfei Han, Xiaodong Li

**Affiliations:** 1 Department of Orthopaedics, The Second People’s Hospital of Changzhi City, No. 83, Heping West Street, Changzhi, 046000 Shanxi China; 2 Graduate School, Changzhi Medical College, No. 161, Jiefang East Street, Changzhi, 046000 Shanxi China; 3 Graduate School, Huazhong University of Science and Technology, No. 13, Hangkong Road, Wuhan, 430000 Hubei China; 4 Department of Orthopaedics, Heping Hospital Affiliated To Changzhi Medical College, No. 110, Yan’an South Road, Changzhi, 046000 Shanxi China

**Keywords:** Total knee arthroplasty, Patellar resurfacing, Patellar non-resurfacing, Denervation

## Abstract

**Background:**

The primary management modalities for the patella in TKA include patellar resurfacing, patellar non-resurfacing, patellar resurfacing with denervation, and patellar non-resurfacing with denervation. Traditionally, meta-analyses have predominantly focused on examining comparisons between two management modalities. However, this study performed a network meta-analysis to compare all four patellar management interventions to identify the most optimal approach for patellar management in TKA.

**Methods:**

A computer-based search of PubMed, China National Knowledge Infrastructure (CNKI), The Cochrane Library, Web of science, Embase, and MEDLINE databases was performed to identify randomized controlled trials focusing on the four management interventions for the patella in TKA. Comparisons included two-by-two comparisons as well as those involving more than two concurrent comparisons. The search timeframe spanned from inception to June 30, 2023. Two independent authors extracted the data and evaluated the quality of the literature. The Cochrane Collaboration Risk of Bias (ROB) tool was used to evaluate the overall quality of the literature. Subsequently, a network meta-analysis was conducted using the “gemtc” package of the R-4.2.3 software. Outcome measures such as anterior knee pain (AKP), reoperation rate, and patient satisfaction rate were evaluated using odd ratio (OR) and 95% confidence intervals (CI). Additionally, the knee society score (KSS), function score (FS), and range of motion (ROM) were evaluated using mean differences (MD) with associated 95% CI. The different treatment measures were ranked using the surfaces under the cumulative ranking curves (SUCRA).

**Results:**

A total of 50 randomized controlled trials involving 9,283 patients were included in the analysis. The findings from this network meta-analysis revealed that patellar resurfacing exhibited significantly lower postoperative reoperation rate (OR 0.44, 95% CI 0.24–0.63) and AKP (OR 0.58, 95% CI 0.32–1) compared to non-resurfacing. Additionally, patellar resurfacing exhibited higher postoperative KSS clinical scores in comparison with non-resurfacing (MD: 1.13, 95% CI 0.18–2.11). However, for postoperative FS, ROM, and patient satisfaction, no significant differences were observed among the four management interventions.

**Conclusion:**

Patellar resurfacing emerges as the optimal management modality in primary TKA. However, future studies should aim to reduce sources of heterogeneity and minimize the influence of confounding factors on outcomes.

**Systematic review registration:**

https://www.crd.york.ac.uk/prospero/display_record.php?ID=CRD42023434418 identifier: CRD42023434418

## Introduction

Total knee arthroplasty (TKA) is an effective treatment for end-stage knee osteoarthritis (KOA) [1]. However, anterior knee pain (AKP) may occur in some patients following primary TKA, with an incidence ranging from 5 to 10%, despite advancements in prosthesis design [2]. Contributing factors to AKP include patellofemoral joint instability, poor patellofemoral joint trajectory, patella baja, and patellar impingement [3, 4]. Therefore, the management of the patella in TKA is of particular significant. Nevertheless, the discrepancies surrounding the management of patella in primary TKA persist, and the main management modalities in current clinical use include patellar resurfacing, patellar non-resurfacing, patellar resurfacing with denervation, and patellar non-resurfacing with denervation. Advocates of patellar resurfacing argue for its superior cost-effectiveness, lower reoperation rates, and reduced incidence of AKP. Conversely, opponents of patellar resurfacing cite higher risks of patellar fracture, patellar dislocation, tendon injury, and patellofemoral popping [5]. They prefer non-resurfacing with bone resection or patellar repair during the procedure, asserting that it preserves enough patellar bone mass and can be easily converted to resurfacing if postoperative recurrent AKP occurs [6]. Additionally, non-resurfacing has been associated with a significant increase in patellar bone density and improved knee function scores due to the preservation of sufficient patellar bone volume [7]. Considering the abundant distribution of neurons around the patella, theoretically, destruction and removal of these pain receptors through an electric knife during TKA may potentially reduce the incidence of postoperative AKP [8], a supposition supported by relevant clinical studies [9]. Therefore, patellar resurfacing or non-resurfacing is often combined with peripatellar denervation during clinical operations. Currently, different modalities for patellar management in primary TKA are employed, and often a combination of these modalities is also used clinically. Thus, determining the optimal treatment remains to be determined. Although classical meta-analysis has been used for such comparisons, its limitation in evaluating two treatments at a time prompted the use of network meta-analysis in this study. This approach combines studies of different management modalities into a network of evidence, allowing for the weighted and combined results of direct and indirect comparisons, quantification, and ranking through surfaces under the cumulative ranking curves (SUCRA). To the best of our knowledge, this is the first study comparing and ranking the efficacy of the four modalities for patellar management commonly used in clinical practice in primary TKA. This network meta-analysis provides insights into the most optimal modality of patellar management in the primary TKA and serves as a reference for intraoperative decision making.

## Materials and methods

This network meta-analysis adheres to the reporting guidelines outlined in PRISMA, which does not require patient agreement and ethical reviews, given that all studies included in the analysis were derived from published research data. We submitted our registration protocol on June 11, 2023, and on June 22, 2023, it was successfully registered. The protocol for this network meta-analysis is available in the International Prospective Register of Systematic Reviews (PROSPERO) (CRD42023434418).

### Search strategy

The databases of PubMed, China National Knowledge Infrastructure (CNKI), The Cochrane Library, Web of science, Embase, and MEDLINE databases were searched for randomized controlled trials on patellar management in primary TKA from inception to June 30, 2023. The search strategy employed for PubMed was: (((“Arthroplasty, Replacement, Knee”[Majr]) OR (((((((((((((((((((((((((((((((Arthroplasties, Replacement, Knee[Title/Abstract]) OR (Arthroplasty, Knee Replacement[Title/Abstract])) OR (Knee Replacement Arthroplasties[Title/Abstract])) OR (Knee Replacement Arthroplasty[Title/Abstract])) OR (Replacement Arthroplasties, Knee[Title/Abstract])) OR (Knee Arthroplasty, Total[Title/Abstract])) OR (Arthroplasty, Total Knee[Title/Abstract])) OR (Total Knee Arthroplasty[Title/Abstract])) OR (Replacement, Total Knee[Title/Abstract])) OR (Total Knee Replacement[Title/Abstract])) OR (Knee Replacement, Total[Title/Abstract])) OR (Knee Arthroplasty[Title/Abstract])) OR (Arthroplasty, Knee[Title/Abstract])) OR (Arthroplasties, Knee Replacement[Title/Abstract])) OR (Replacement Arthroplasty, Knee[Title/Abstract])) OR (Arthroplasty, Replacement, Partial Knee[Title/Abstract])) OR (Unicompartmental Knee Arthroplasty[Title/Abstract])) OR (Arthroplasty, Unicompartmental Knee[Title/Abstract])) OR (Knee Arthroplasty, Unicompartmental[Title/Abstract])) OR (Unicondylar Knee Arthroplasty[Title/Abstract])) OR (Arthroplasty, Unicondylar Knee[Title/Abstract])) OR (Knee Arthroplasty, Unicondylar[Title/Abstract])) OR (Partial Knee Arthroplasty[Title/Abstract])) OR (Arthroplasty, Partial Knee[Title/Abstract])) OR (Knee Arthroplasty, Partial[Title/Abstract])) OR (Unicondylar Knee Replacement[Title/Abstract])) OR (Knee Replacement, Unicondylar[Title/Abstract])) OR (Partial Knee Replacement[Title/Abstract])) OR (Knee Replacement, Partial[Title/Abstract])) OR (Unicompartmental Knee Replacement[Title/Abstract])) OR (Knee Replacement, Unicompartmental[Title/Abstract]))) AND ((“Patella”[Majr]) OR (((((Patellas[Title/Abstract]) OR (Kneecap[Title/Abstract])) OR (Kneecaps[Title/Abstract])) OR (Knee Cap[Title/Abstract])) OR (Knee Caps[Title/Abstract])))) AND (((clinical[Title/Abstract]) AND (trial[Title/Abstract])) OR (((“Clinical Trials as Topic”[Majr]) OR “Clinical Trial” [Publication Type]) OR “Random Allocation”[Majr])).

### Inclusion and exclusion criteria

The inclusion criteria for the studies included in the network meta-analysis were the following: (1) studies employing randomized controlled trials; (2) studies that included two or more of the four management interventions (patellar resurfacing, non-resurfacing, patellar resurfacing with denervation, and non-resurfacing with denervation); (3) studies with follow-up results, with a minimum follow-up period of 6 months; (4) the chosen outcome indicators for the study included AKP, reoperation, KSS, FS, ROM, and patient satisfaction; (5) studies that involved patients who underwent primary TKA.

### Data extraction

Two independent researchers conducted data extraction from the original paper. In instances where discrepancies arose between the two individual researchers during the processing the data, consultation with a third researcher (senior chief physician) was sought for the final decision. The extracted data included (1) authors; (2) year of publication; (3) location of the study; (4) sample size; (5) mean age; (6) gender; (7) mean follow-up time; (8) outcome indicators, including AKP, reoperation, KSS, FS, ROM, and patient satisfaction.

### Quality assessment

The assessment of the inclusion of randomized controlled trials underwent quality evaluation using the Cochrane Risk of Bias (ROB) assessment tool. The tool incorporates seven key items for assessment, namely, randomized sequence generation, allocation concealment, blinding of patients and physicians, blinding of outcome assessors, incomplete outcome data, selective reporting of study results, and other biases.

### Statistical analysis

A Bayesian-based network meta-analysis was performed using the “gemtc” package in R-4.2.3 software. The Bayesian Markov chain Monte Carlo random-effects model was employed for sampling simulation and calculation. Specifically, the Markov chain was set to 4, with 50,000 iterations. The initial 20,000 iterations were used for annealing to eliminate the effect of the initial values, whereas the subsequent 30,000 iterations were used for sampling, with an iteration step of 1. To ensure the validity of the analysis, convergence diagnostic plots, trace plots, and density plots were generated. The potential scale reduction factors (PSRF) were employed to assess iterative convergence, with a target range set at 1–1.05 for satisfactory convergence. The inconsistency test was performed using the node-splitting method, with a non-statistically significant difference (*P* > 0.05) between direct and indirect comparison results. Heterogeneity was assessed using the I2 statistics, where *I*^2^ ≤ 50% suggested no heterogeneity, prompting the selection of a fixed-effects model. Conversely, *I*^2^ > 50% indicated the presence of heterogeneity, requiring further analysis to identify and address potential sources. If heterogeneity persisted, and clinical consistency was maintained, then a random-effects model was applied. Moreover, we performed sensitivity analysis that involved iteratively excluding one literature piece at a time and analyzing the remaining studies to determine the impact on the results. The ORs with 95% CIs were calculated for each intervention for dichotomized variables, considering statistical significance for CIs not including 1. For continuous variables, the MD with 95% CIs were assessed, with statistical significance attributed to CIs not containing 0. The SUCRA values were calculated and ranked using the “gemtc” package in R software to determine optimal treatments, and ranking was plotted for visual presentation.A comparison-correction funnel plot was generated in Stata 17.0 to assess publication bias. Symmetrical distribution around the effect points of independent studies indicated the absence of publication bias (i.e., the icon showed an inverted symmetrical funnel shape), whereas publication bias was considered for asymmetrical distribution. The degree of asymmetry or incompleteness was used to reflect the magnitude of publication bias.

## Results

### Study characteristics

**Fig. 1:**
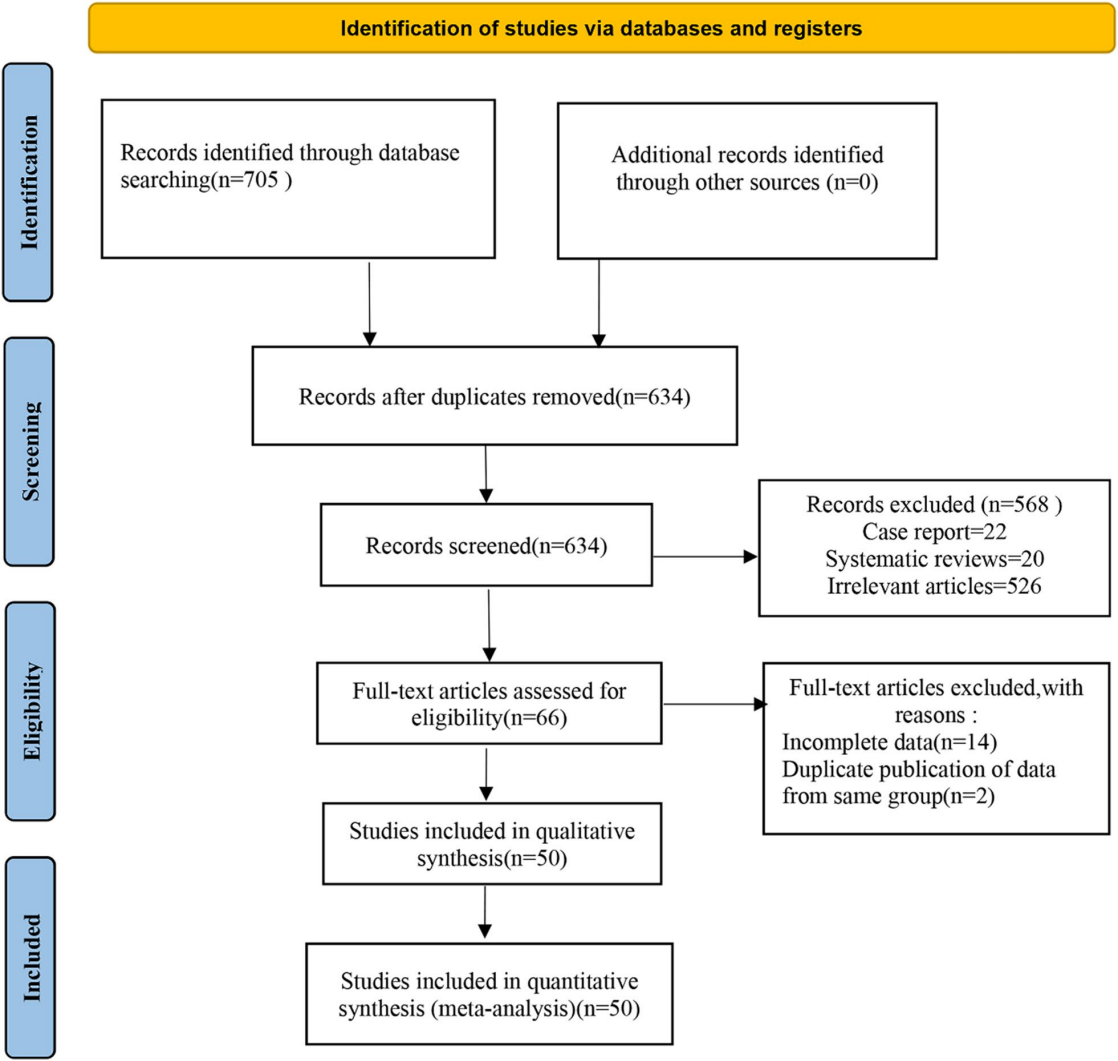
The flow diagram of study selection

The search flowchart is illustrated in Fig. 1. We searched PubMed, China National Knowledge Infrastructure (CNKI), The Cochrane Library, Web of science, Embase, and MEDLINE databases and found a total of 705 articles (Fig. 1). Subsequently, all searched papers were imported onto EndnoteX9 software and duplicate papers (*n* = 71) were excluded. Furthermore, 568 papers were excluded after review of the titles and abstracts. After the initial screening, 66 papers were identified as suitable for the topic of this study; however, 16 papers were further excluded after review of their contents and outcome indicators. Ultimately, a total of 50 randomized controlled trials, involving 9283 patients, were included in the final analysis (Fig. 1).

Before the surgical procedure, no statistically significant difference in sample size, mean age, and sex ratio was observed between the two groups (Table 1). All included papers met our pre-set inclusion criteria, and each randomized controlled trial included two of the four interventions for comparison. Furthermore, the mean follow-up time for all included studies was at least 6 months or longer.

**Table 1:** Characteristics of included literature studies

References	Country	Treatment	Sample size	Age (years)	Sex (woman, %)	Mean follow-up (months)	Outcome
Bourne [10]	USA	A	50	72 ± 7	42	24	RP, FS
		B	50	68 ± 7			
Feller [11]	Australia	A	19	70.5 ± 6.6	36.8	39.1 ± 2.5	RP
		B	19	71.1 ± 5.6		37.8 ± 2.4	
Barrack [12]	USA	A	42	65.3	20.9	30	RP, AKP, KSS, Satisfaction
		B	44	67.1			
Kajino [13]	Japan	A	26	56.1	92.3	79.2	RP
		B	26				
Schroeder-Boersh [14]	Germany	A	20	73	70	24	RP, KSS, FS
		B	20	72.2			
Newman [15]	UK	A	42	72	64.6	60	RP, AKP
		B	42	71.2			
Barrack [16]	USA	A	47	66.2	NA	60–84	RP, AKP, KSS, FS, Satisfaction
		B	46				
Wood [17]	Australia	A	92	73.7	47.3	48	RP, AKP, KSS, FS, Satisfaction
		B	128				
Mayman [18]	Canada	A	50	72 ± 7	42	120	RP, Satisfaction
		B	50	68 ± 7			
Waters [19]	UK	A	243	69.1	NA	63.6	RP, AKP, KSS, FS, Satisfaction
		B	231				
Burnett [20]	Canada	A	42	71 ± 8	56.7	120	RP, AKP, KSS, FS, ROM, Satisfaction
		B	48	69 ± 8			
Gildone [21]	Italy	A	28	73.6	69.6	24	AKP, FS, ROM
		B	28	74.6			
Campbell [22]	Australia	A	46	71	72	120	RP, AKP, KSS
		B	54	73			
Myles [23]	UK	A	25	70	48	24	KSS, FS
		B	25				
Smith [24]	Australia	A	24	72 ± 7	46.3	NA	AKP
		B	17	68 ± 7			
Burnett [25]	USA	A	28	78	NA	120	RP, AKP, KSS, FS, ROM, Satisfaction
		B	28				
Liu [26]	China	A	30	68	81.7	54	AKP, FS, ROM, Satisfaction
		B	30				
Smith [27]	Australia	A	87	71.9	50.3	52.44	RP, AKP, KSS, FS, Satisfaction
		B	94	71.2			
Burnett [28]	USA	A	58	65.3	NA	120	RP, AKP, KSS, FS, ROM, Satisfaction
		B	60	67.1			
Breeman [29]	UK	A	861	70	NA	60	RP
		B	854	70			
Van jonbergen [8]	Netherland	B	131	72	68.3	12	RP, AKP, KSS, FS
		D	131	71			
Altay [9]	Turkey	B	35	68	74.3	36	KSS, FS, ROM
		D	35				
Baliga [30]	UK	B	94	69.2	49.7	12	AKP
		D	91	69			
Beaupre [31]	Canada	A	21	64.9 ± 4	68.4	120	RP
		B	17	62 ± 5.6			
Liu [6]	China	A	68	67.5 ± 7.2	37.1	84	RP, AKP, KSS, FS, Satisfaction
		B	64	68 ± 6.7			
Sun [32]	China	B	76	64.2	52.6	55	AKP, KSS, FS, ROM, Satisfaction
		D	76	65.1			
Bao [33]	China	A	32	67	77.8	12	AKP, KSS, FS
		D	31	66			
Ferguson [34]	UK	A	88	69.8 ± 8.2	53.4	24	RP, KSS, FS, ROM
		B	88				
Ferguson [34]	UK	A	89	70.2 ± 7.6	52.8	24	RP, KSS, FS, ROM
		B	87				
Murray [35]	UK	A	861	70	55.7	120	RP
		B	854				
Pulavarti [36]	UK	B	63	69.8	54	26	KSS, FS, ROM, Satisfaction
		D	63	69.9			
Sreehari [37]	UK	A	75	68.1	NA	60	RP
		B	60	65.8			
Alomran [38]	Saudi Arabia	B	92	NA	NA	24	AKP, ROM, Satisfaction
		D	92				
Kwon [39]	Korea	B	50	67 ± 3.7	NA	60	KSS
		D	50	66.3 ± 3.5			
Roberts [40]	USA	A	178	70.2 ± 8.7	51.4	124.8	RP, AKP, KSS, FS, ROM
		B	172	71.3 ± 7.4			
Ali [41]	Sweden	A	35	68 ± 4	60.8	72	Satisfaction
		B	39	69 ± 4			
Aunan [42]	Norway	A	63	70	56.6	36	RP, KSS, FS
		B	66	69			
Vukadin [43]	Serbia	A	30	68.1 ± 7.0	55	24	RP, KSS, FS
		B	30	66 ± 6.4			
Wang [44]	China	A	14	66.9 ± 7.8	100	12	AKP, KSS, ROM
		D	14				
Dong [45]	China	C	48	67.7 ± 6.2	57.3	36	RP, AKP, KSS, FS, Satisfaction
		D	48				
Kaseb [46]	Iran	A	24	64.8 ± 7.8	84	6	KSS, FS
		D	26				
Ha [47]	China	A	60	65.2	36.7	60	AKP, KSS, FS, Satisfaction
		B	60				
Kaseb [48]	Iran	A	29	68.1 ± 7.7	79.5	8.68	KSS, FS
		B	44	65.8 ± 6.9			
Koh [49]	Korea	A	49	70 ± 5.7	98	60	AKP, Satisfaction
		B	49				
Thiengwittayaporn [50]	Thailand	A	41	68.2 ± 8.2	82.5	12	AKP, KSS, ROM
		B	39	68.2 ± 8.0			
Budhiparama [51]	Indonesia	B	73	66 ± 7	91.8	30 ± 5.9	ROM
		D	73				
Goicoechea [52]	Spain	A	81	72.7 ± 8.2	70.4	12	KSS, FS
		C	88				
Raaij [53]	Netherland	C	21	67.3 ± 8.6	61.9	24	RP, AKP, KSS, FS
		D	21	71.6 ± 8.0			
Thiengwittayaporn [54]	Thailand	A	111	69.4 ± 6.9	81	24	KSS, ROM
		C	110	69.2 ± 7.2			
Deroche [55]	France	A	118	68.8 ± 7.8	58.1	17.6	RP, KSS, FS, ROM
		D	111	69.7 ± 8.3			
Cankaya [56]	Turkey	A	25	68.4 ± 7.6	86	12	KSS, FS, ROM
		B	25	71.8 ± 8.4			

A: patellar resurfacing, B: patellar non-resurfacing, C: patellar resurfacing with denervation, D: patellar non-resurfacing with denervation

*RP* reoperation, *AKP* anterior knee pain, *KSS* knee society score, *FS* function score, *ROM* range of motion

### Methodological quality

**Fig. 2:**
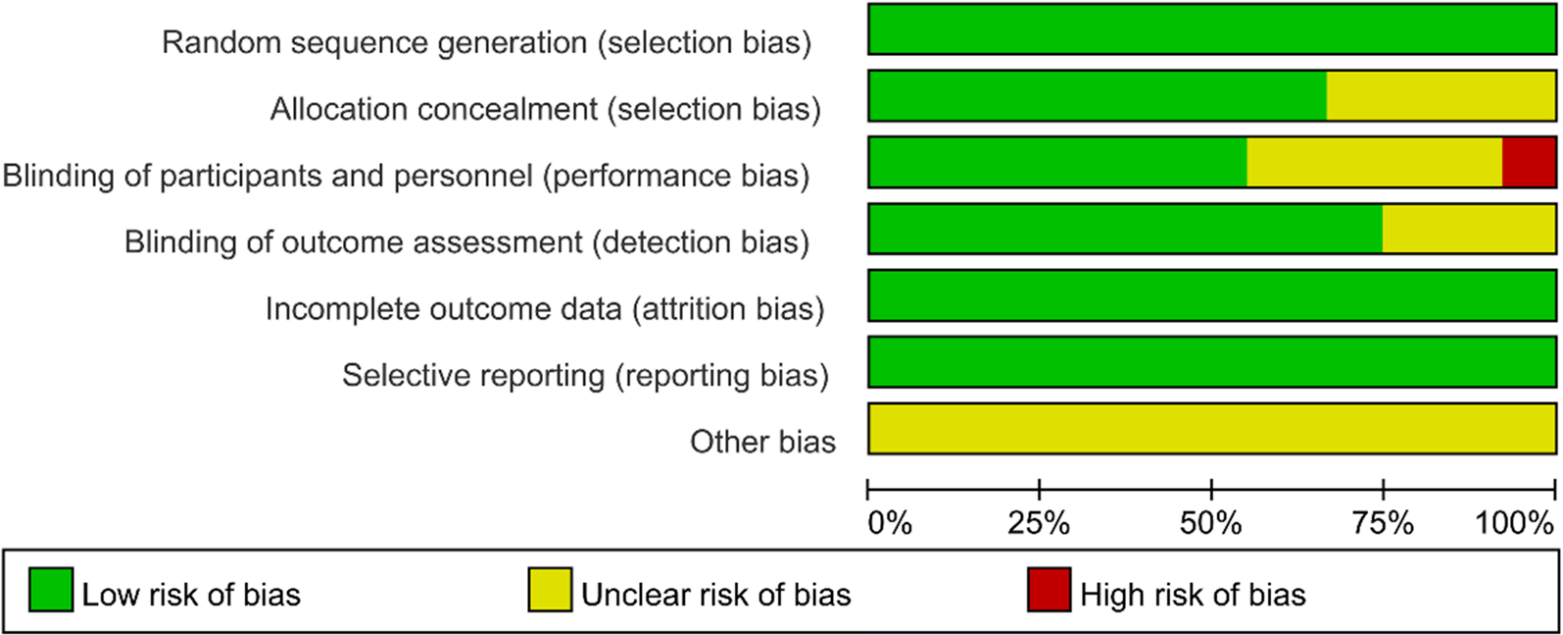
Risk of graph of the included studies

**Fig. 3:**
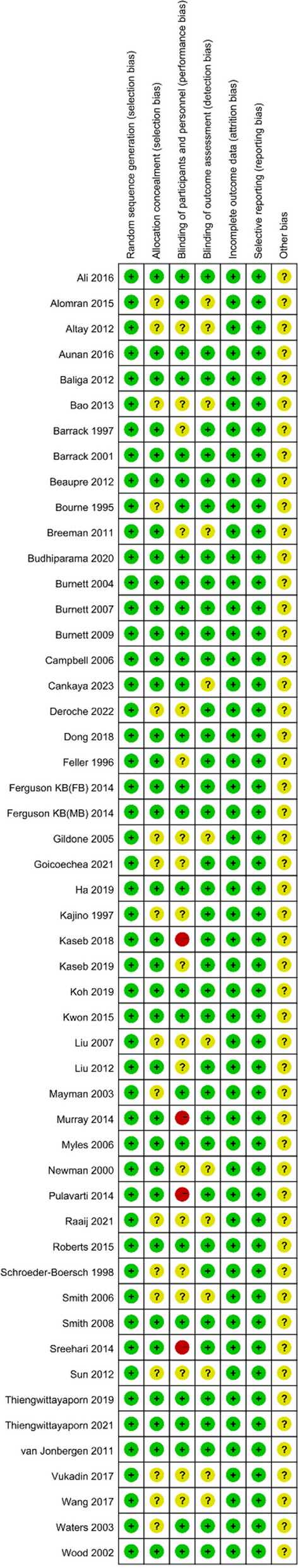
Risk of bias assessment of included studies

The results of the ROB assessment are presented in Figs. 2 and 3. In terms of random allocation, all studies reported the method of random allocation, indicating a low ROB in this aspect. For allocation concealment, 17 studies did not specify the method of allocation concealment [9, 10, 13, 14, 18, 19, 21, 24, 26, 32, 33, 38, 43, 44, 52, 53, 55], resulting in uncertain ROB. For double blinding, due to the inherent nature of the procedure, four studies [35–37, 46] acknowledged that patients undergoing the procedure were unavoidably aware of what they were to experience, leading to a high ROB. In 19 studies [6, 9, 11–15, 21, 24, 26, 29, 32, 33, 43, 44, 48, 52, 53, 55], double blinding was not explicitly specified, resulting in indeterminate risk. For blinding of outcome assessors, 13 studies [9, 15, 21, 24, 26, 29, 32, 33, 38, 43, 44, 53, 56] did not explicitly indicate their methods, leading to an uncertain ROB. Furthermore, completeness of results and selective reporting were deemed to be at low ROB. The assessment of other biases yielded uncertain risk.

## Results of network meta-analysis

### Maps of network evidence

**Fig. 4:**
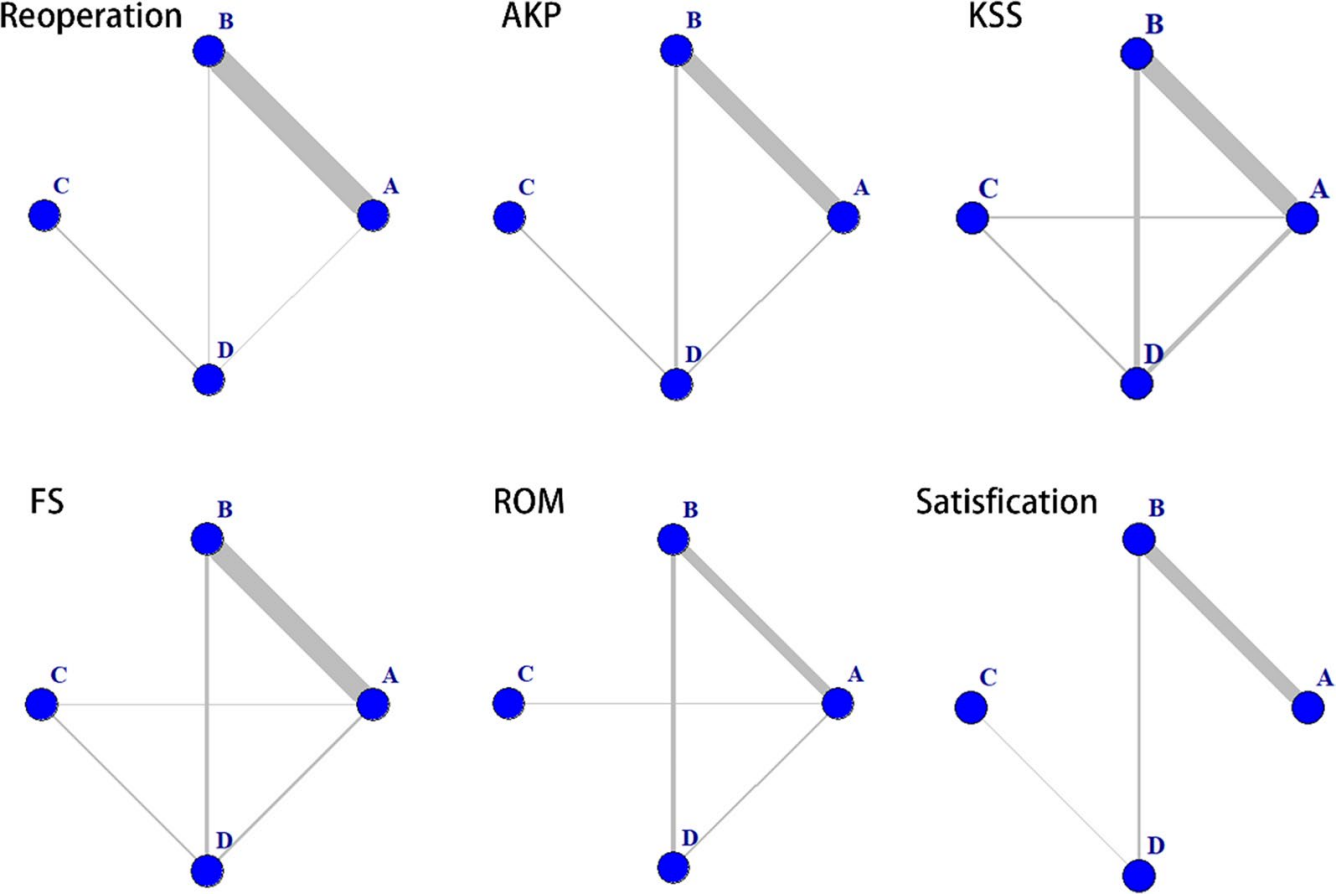
Network evidence maps. A: Patellar resurfacing, B: patellar non-resurfacing, C: patellar resurfacing with denervation, D: patellar non-resurfacing with denervation. *RP* reoperation, *AKP* anterior knee pain, *KSS* knee society score, *FS* function score, *ROM* range of motion

The “gemtc” package in R software was employed to generate a network evidence graph, representing the four modes of intervention. Figure 4 illustrates the network evidence graph, and the lines between the nodes represent the number of directly comparable interventions, with the thickness of the lines indicating the frequency of two-by-two comparisons.

### Heterogeneity and consistency test results

**Fig. 5:**
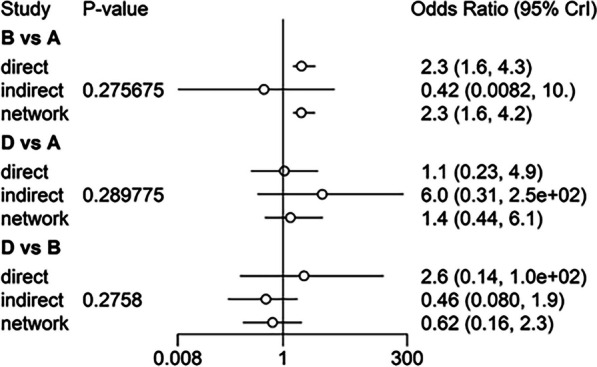
Comparison between direct and indirect evidence—RP

**Fig. 6:**
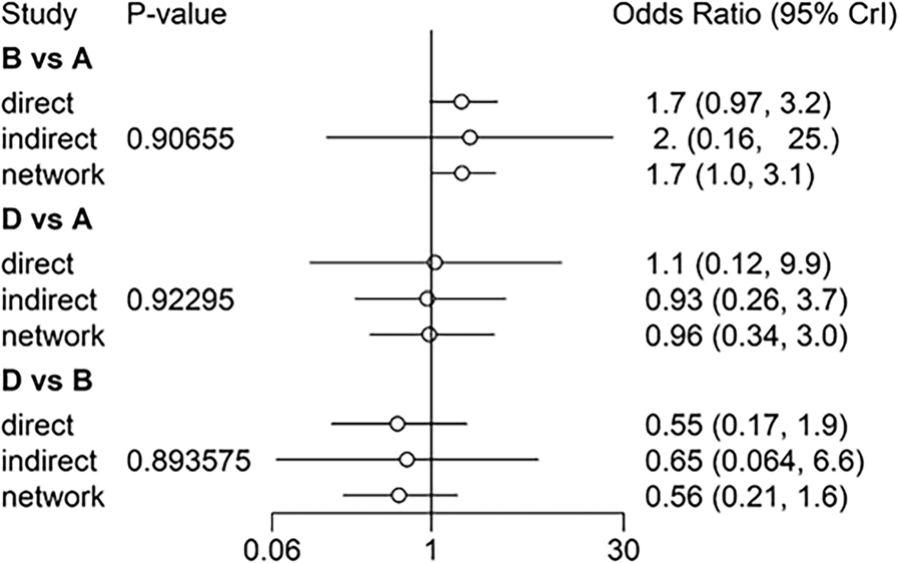
Comparison between direct and indirect evidence—AKP

**Fig. 7:**
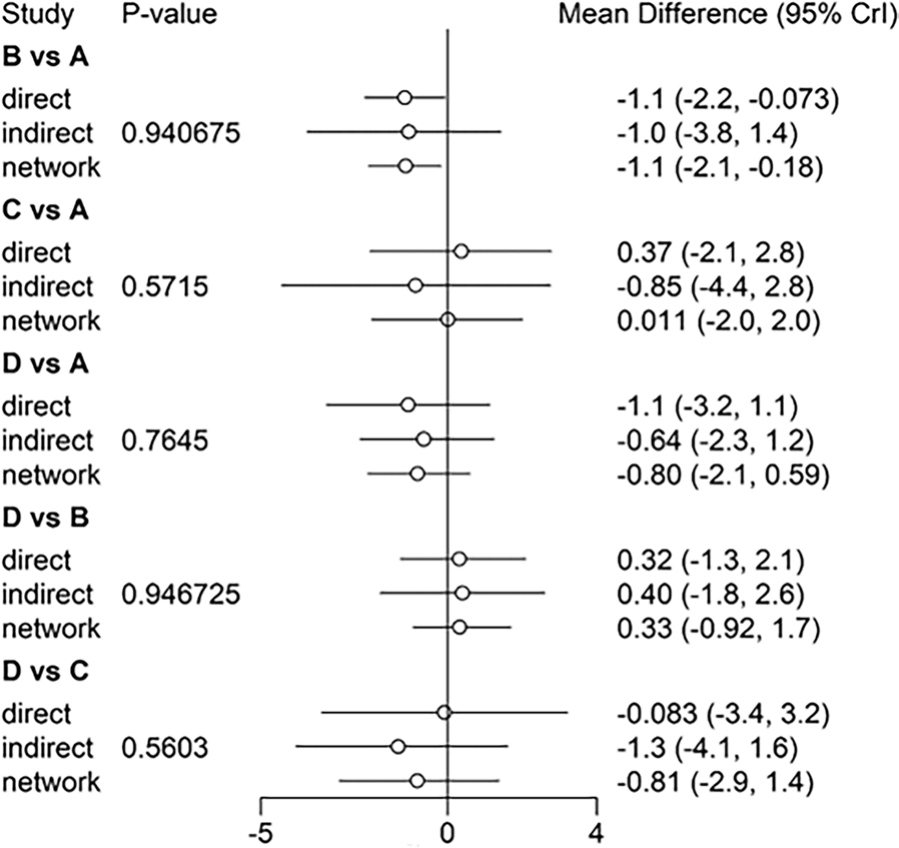
Comparison between direct and indirect evidence—KSS

**Fig. 8:**
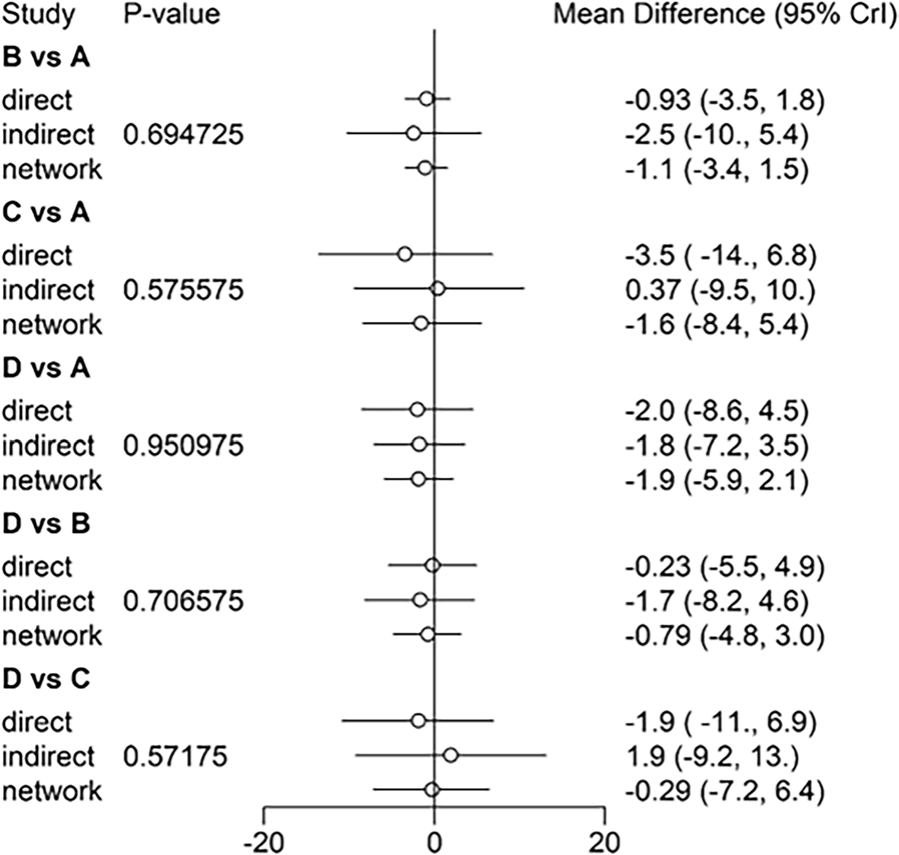
Comparison between direct and indirect evidence—FS

**Fig. 9:**
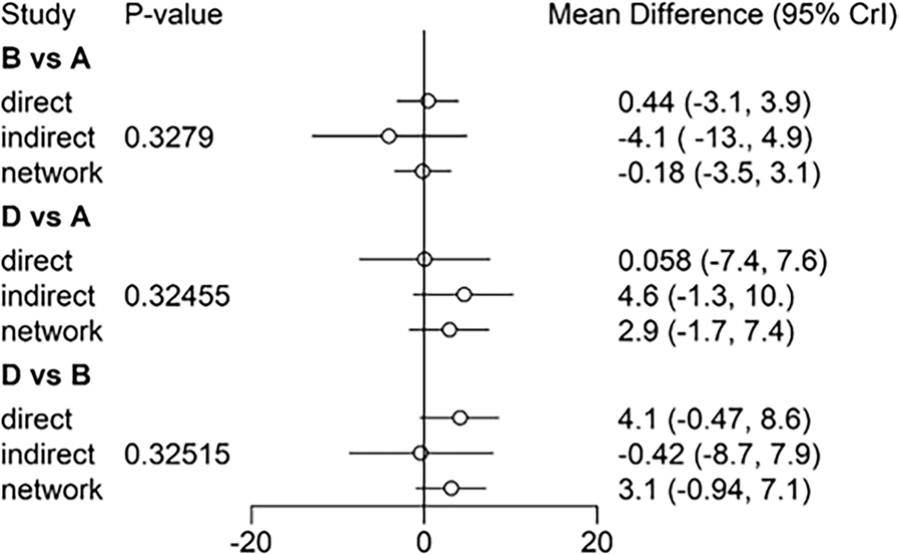
Comparison between direct and indirect evidence—ROM

Heterogeneity test of classical meta-analysis was performed on the included studies, revealing *I*^2^ > 50%. Consequently, the random-effects model was used for the analysis. The consistent and inconsistent models were fitted separately, and the comparison of the deviance information criterion (DIC) indicated that the DIC values for both models were larger, with a difference of < 5. This implies that the two models were fitted to a similar degree, suggesting stable results that could be reliably analyzed using the consistent model. To further assess for local inconsistency, the node-splitting method was applied. The results revealed *P* > 0.05, indicating that local inconsistency between studies might be small. Therefore, the network meta-analysis was performed under the consistency model. The direct and indirect evidence for each outcome indicator is shown in Figs. 5, 6, 7, 8, and 9. Since the network evidence map for satisfaction (Fig. 4) did not close the loop, no further test for inconsistency was needed.

### Results of the convergence evaluation

The convergence and stability of each Markov chain were assessed through 20,000 iterations. The iteration trace demonstrated a stable level, indicating that the model had stable convergence. Additionally, as the number of iterations reached 50,000, the bandwidth tended toward 0 and achieved stability, suggesting that the model was of favorable convergence. Furthermore, the Brooks–Gelman–Rubin diagnostics revealed the intermediate value of the PSRF and the 97.5% quantile convergence between 30,000 and 50,000 iterations, ultimately converging to 1. The scale reduction factor reaching 1 is suggestive of satisfactory convergence of the model.

### Results of outcome indicator

#### RP

**Fig. 10:**
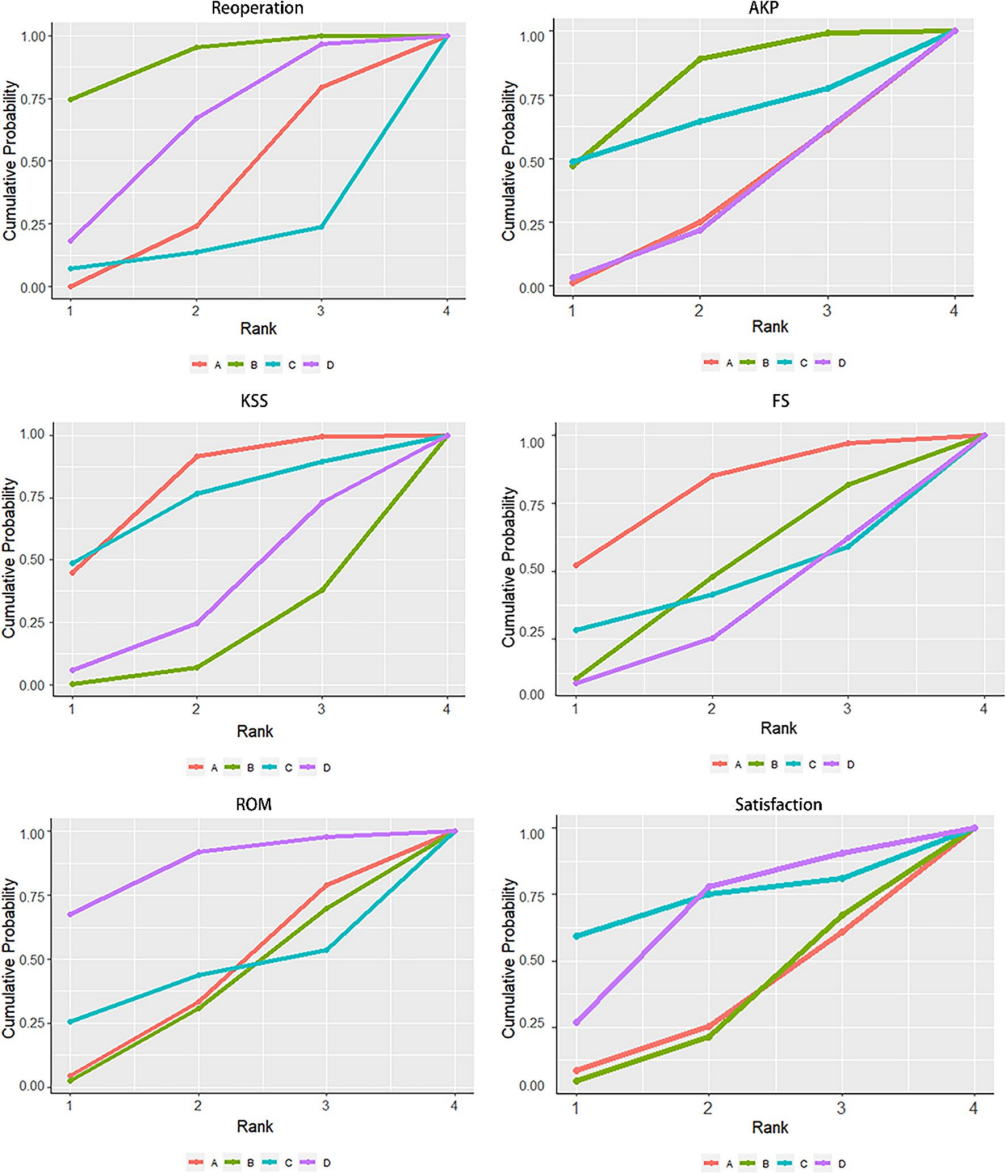
Surfaces under the cumulative ranking curves (SUCRA) for reoperation, anterior knee pain, knee society score, function score, range of motion, satisfaction. The graph displays the distribution of probabilities for each treatment. The X-axis represents the rank, and the Y-axis represents probabilities

A total of 29 studies reported reoperation, and the results showed that patellar resurfacing had a significantly lower postoperative reoperation compared to patellar non-resurfacing (OR 0.44, 95% CI 0.24–0.63). The other differences did not display significance and are outlined in Table 2. The results of the SUCRA ranking, from worst to best, were patellar non-resurfacing (0.9) > patellar non-resurfacing with denervation (0.606) > patellar resurfacing (0.345) > patellar resurfacing with denervation (0.149) (Fig. 10).

**Table 2:** League tables between two-by-two comparisons

RP	**A**			
	*0.44(0.24,0.63)*	**B**		
	3.01(0.17,126.32)	7.11(0.42,312.88)	**C**	
	0.7(0.16,2.25)	1.6(0.41,5.87)	0.23(0.01,2.8)	**D**
AKP	**A**			
	*0.58(0.32,1)*	**B**		
	0.59(0.05,5.06)	1.01(0.09,8.44)	**C**	
	1.04(0.33,3.01)	1.79(0.63,4.85)	1.77(0.27,15.63)	**D**
KSS	**A**			
	*1.13(0.18,2.11)*	**B**		
	− 0.01(− 2.0,2.0)	− 1.14(− 3.3,0.96)	**C**	
	0.8(− 0.59,2.12)	− 0.33(− 1.7,0.92)	0.82(− 1.4,2.90)	**D**
FS	**A**			
	1.11(− 1.50,3.44)	**B**		
	1.61(− 5.49,8.40)	0.50(− 6.64,7.53)	**C**	
	1.90(− 2.10,5.90)	0.79(− 3.02,4.83)	0.29(− 6.40,7.20)	**D**
ROM	**A**			
	0.18(− 3.10,3.50)	**B**		
	0.30(− 9.7,10.26)	0.13(− 10.44,10.6)	**C**	
	− 2.96(− 7.44,1.70)	− 3.13(− 7.10,0.94)	− 3.26(− 14.10,7.86)	**D**
Satisfaction	**A**			
	0.99(0.51,1.98)	**B**		
	0.41(0.03,6.24)	0.41(0.03,5.71)	**C**	
	0.58(0.13,2.61)	0.58(0.16,2.21)	1.43(0.15,13.89)	**D**

A: patellar resurfacing, B: patellar non-resurfacing, C: patellar resurfacing with denervation, D: patellar non-resurfacing with denervation

*RP* reoperation, *AKP* anterior knee pain, *KSS* knee society score, *FS* function score, *ROM* range of motion. Italics were with statistically significant

#### AKP

A total of 27 studies reported postoperative AKP, and the results showed that the postoperative AKP of patellar resurfacing was lower than that of patellar non-resurfacing, and the difference was significant [OR 0.58,95% confidence interval (0.32,1)], and the other differences were not significant, as shown in Table 2. The results of the SUCRA ranking, from worst to best, were patellar non-resurfacing (0.785) > patellar resurfacing with denervation (0.636) > patellar resurfacing (0.291) > patellar non-resurfacing with denervation(0.288) (Fig. 10).

#### KSS

A total of 30 studies reported KSS clinical scores, and the results showed that the postoperative KSS clinical scores of patellar resurfacing were higher than those of patellar non-resurfacing, and the difference was significant [MD = 1.13, 95% confidence interval (0.18, 2.11)], and the other differences were not significant, as shown in Table 2. The results of the SUCRA ranking, from the most favorable to the most unfavorable, were patellar resurfacing (0.787) > patellar resurfacing with denervation (0.716) > patellar non-resurfacing with denervation (0.346) > non-resurfacing (0.151) (Fig. 10).

#### FS

A total of 31 studies reported KSS functional scores, and the results showed that no difference was significant. The SUCRA ranking results, from best to worst, were patellar resurfacing (0.780) > patellar non-resurfacing (0.468) > patellar resurfacing with denervation (0.430) > patellar non-resurfacing with denervation (0.322), as shown in Fig. 10.

#### ROM

A total of 15 research studies reported ROM, and the results showed that no differences were significant. The SUCRA ranking results, from best to worst, were patellar non-resurfacing with denervation (0.860) > patellar resurfacing with denervation (0.410) > patellar resurfacing (0.390) > patellar non-resurfacing (0.343), as shown in Fig. 10.

#### Satisfaction

A total of 18 studies reported patient satisfaction, and no differences were significant. SUCRA ranking results, from best to worst, were patellar resurfacing with denervation (0.820) > patellar non-resurfacing with denervation (0.651) > patellar resurfacing (0.317) > patellar non-resurfacing (0.313), as shown in Fig. 10.

#### Risk of publication bias analysis of included studies

**Fig. 11:**
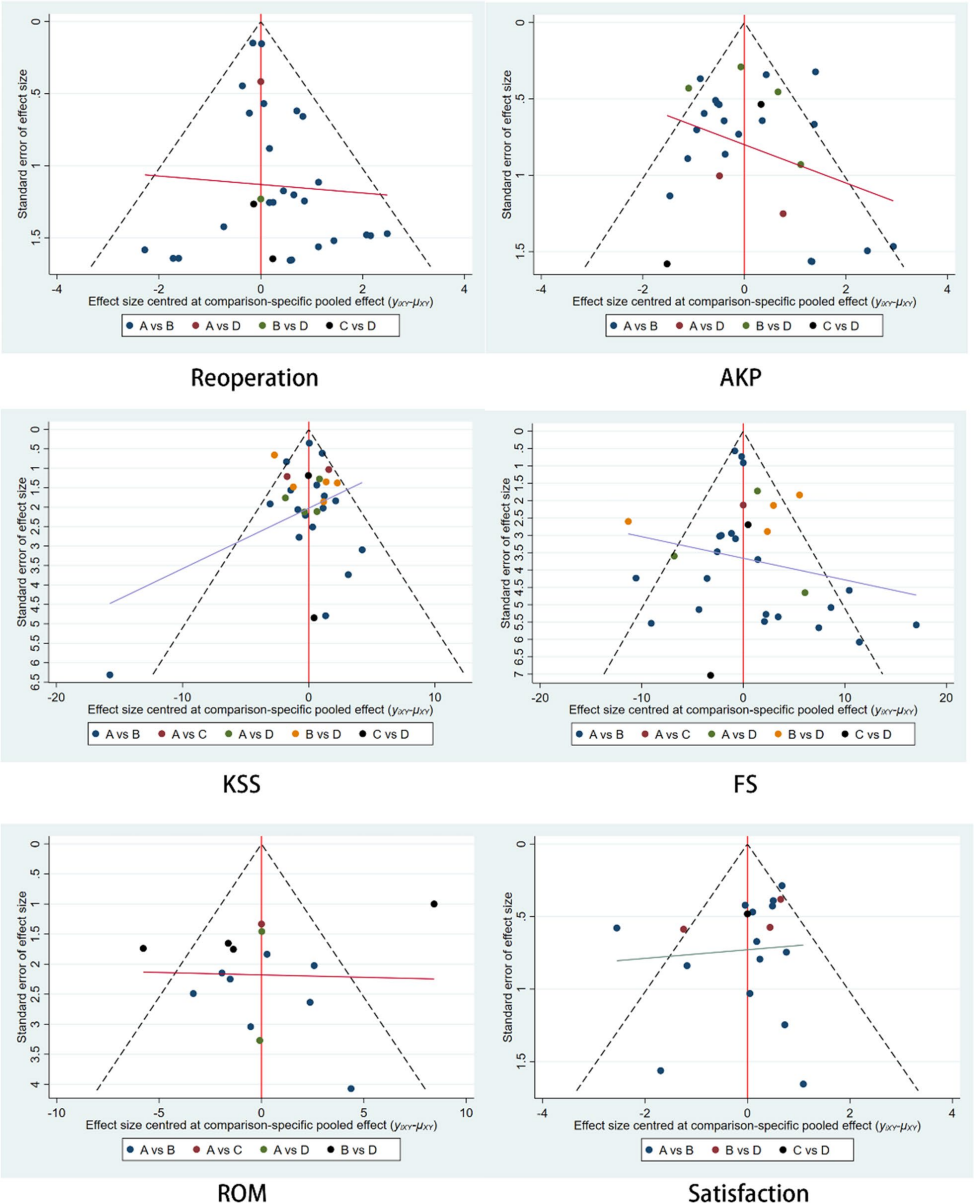
Comparison-correction funnel plot

Publication bias was analyzed for each outcome indicator by Stata17.0 software, and the plotted comparison-correction funnel plots are shown in Fig. 11, which showed that the funnel plots were basically symmetrical, suggesting that there was no significant publication bias.

## Discussion

In this network meta-analysis based on 50 randomized controlled trials, we compared four management modalities for the patella in primary TKA: patellar resurfacing, patellar non-resurfacing, patellar resurfacing with denervation, and patellar non-resurfacing with denervation. Patellar resurfacing was the most satisfactory management of the patella in primary TKA, with significantly lower AKP and reoperation after patellar resurfacing than patellar non-resurfacing, and significantly higher postoperative KSS scores than patellar non-resurfacing. There were no significant differences between these management modalities in terms of postoperative FS, ROM, or patient satisfaction. For whether to combine peripatellar denervation in patellar resurfacing or patellar non-resurfacing, our results showed no significant difference. These results may help joint surgeons to choose the most suitable patellar management when operating on patients undergoing primary TKA.

This is a first network meta-analysis comparing the four modalities of management commonly used for the patella in primary TKA. There is still controversy about whether to perform patellar resurfacing, and in the prior study, Chen [57] compared patellar resurfacing with patellar non-resurfacing in primary TKA in a classic meta-analysis, and his results showed that patellar resurfacing reduces reoperation rates and improves the KSS scores and FS scores compared with patellar non-resurfacing, but it may not affect the postoperative AKP, ROM, and patient satisfaction rate, which is close to but different slightly from the conclusion we concluded by Bayesian network meta-analysis. In Parsons' meta-analysis [58], reoperation rates were lower for patellar resurfacing than for non-patellar resurfacing. In Grela's meta-analysis [59], patellar resurfacing reduced postoperative AKP and reoperation rates, whereas there was no significant difference in postoperative ROM, which is consistent with our results. In a network meta-analysis by Arirachakaran comparing patellar resurfacing, patellar non-resurfacing, and peripatellar denervation in primary TKA [60], his study demonstrated that peripatellar denervation significantly reduced postoperative AKP and improved postoperative KSS scores compared to patellar resurfacing and non-resurfacing, which was in contrast to our results. Also in his study, patellar resurfacing significantly reduced postoperative reoperation rates compared to patellar non-resurfacing, which is consistent with our results. Whether to combine peripatellar denervation with patellar resurfacing or patellar non-resurfacing is also controversial in clinical situations. In a meta-analysis by Fan [61], it was concluded that peripatellar denervation did not affect the incidence of postoperative AKP, and it could significantly improve the knee function, which was beneficial to the prognosis of primary TKA. In contrast, in Xie's meta-analysis [62], it was concluded that peripatellar denervation did not reduce anterior knee pain or improve clinical prognosis after 12 months of follow-up; nevertheless, Xie recommended peripatellar denervation because of its good safety profile, which was in agreement with our results. In Zhang's meta-analysis [63], his conclusions showed that combining peripatellar denervation improved clinical prognosis, but it was not recommended.

At present, most scholars believe that patellar resurfacing should be performed when there is severe patellofemoral cartilage abrasion and poor patellar alignment, but also indicate that patellar resurfacing is not suitable when the patella is too small. Patellar resurfacing is not recommended for young patients with knee osteoarthritis who have mild or medium damage to the patellofemoral cartilage, or for patients with knee osteoarthritis who have a high level of sports activity. For patients with primary TKA, postoperative AKP can be reduced when the patella is fully apposed to the femoral trochlea carriage. Therefore, intraoperative patellar alignment needs to be determined based on the thumbless test to choose whether or not to perform patellar resurfacing [64], and the degree of wear and tear of the patellar cartilage, patellar size, and thickness also need to be observed to choose the appropriate option. For peripatellar denervation, it can theoretically reduce postoperative AKP due to the reduction of pain receptors in synovial tissue by cauterizing the soft tissues around the patella with an electric knife [38], and it can be considered with a short operation time during surgery. This article is the first network meta-analysis comparing the currently commonly used treatments of the patella in primary TKA, with the inclusion of 50 randomized controlled trials. We compared four treatments concurrently by means of a Bayesian network meta-analysis incorporating direct and indirect evidence from randomized controlled trials. However, this paper also has limitations, such as: (1) there are fewer randomized controlled trials of patellar resurfacing or non-patellar resurfacing combined with circumpatellar denervation, which may affect the final results; (2) some of the RCTs have not been under-reported, and there may be heterogeneity due to the inconsistency of sample size and follow-up time between different RCTs; (3) at the same time, because the patellar shape and patellar height between different races also differ, surgeons in different countries have different choices of patellar management, which may affect the strength of the evidence; (4) there are different types and designs of knee prostheses, such as patella-friendly prostheses and non-patella-friendly prostheses, which may affect the clinical outcomes as well as the results of the present network meta-analysis; (5) some patients may have concurrent patellofemoral osteoarthritis preoperatively, which may affect the final outcome; and (6) there is also variation in the level of surgical skill of surgeons, which again may affect patient prognosis. Therefore, future research should aim to reduce the confounding factors that affect outcomes, such as: (1) the effect of different knee prosthesis types on patellar replacements; (2) the effect of different grades of patellofemoral arthritis on AKP after TKA; and (3) research on different ethnic groups.

## Conclusion

Patellar resurfacing emerges as the optimal patella management in primary TKA. Although this study consists of a large sample size with 50 randomized controlled trials with a low ROB, it is essential to acknowledge the various factors that may influence the interpretation of the results. The study incorporates data from different countries and ethnicities, potentially introducing variability in the outcomes. Furthermore, the exclusion of some studies due to incomplete data reporting could impact the final analysis. Additionally, the variability in the types of prostheses used across different countries and RCTs is another consideration impacting the final results. All these factors could be sources of heterogeneity, ultimately leading to biased results.

## Data Availability

We state that the data will not be shared since all the raw data are present in the figures included in the article.

## Abbreviations

TKA: Total knee arthroplasty
KOA: Knee osteoarthritis
AKP: Anterior knee pain
SUCRA: Surfaces under the cumulative ranking curves
OR: Odds ratio
CI: Confidence interval
MD: Mean difference
MCMC: Markov chain Monte Carlo
PSRF: Potential scale seen factor
